# The Predictor Roles of Spiritual Well-Being, Healthcare Professionals’ Support and Shock Anxiety in Implantable Cardioverter-Defibrillator Device Acceptance

**DOI:** 10.21315/mjms2022.29.3.8

**Published:** 2022-06-28

**Authors:** Nilofar Pasyar, Masoume Rambod, Alireza Zare, Mohammad Hossein Nikoo

**Affiliations:** 1 Community Based Psychiatric Care Research Center, School of Nursing and Midwifery, Shiraz University of Medical Sciences, Shiraz, Iran; 2 School of Nursing and Midwifery, Shiraz University of Medical Sciences, Shiraz, Iran; 3 Student Research Committee, Shiraz University of Medical Sciences, Shiraz, Iran; 4 Cardiovascular Research Center, Cardiology Department, Shiraz University of Medical Sciences, Shiraz, Iran; 5 Non-Communicable Disease Research Center, Shiraz University of Medical Sciences, Shiraz, Iran

**Keywords:** device acceptance, healthcare support, implantable defibrillator, spirituality, shock anxiety

## Abstract

**Background:**

Acceptance of the implantable cardioverter-defibrillator (ICD) device may be affected by a variety of factors. This study aimed to investigate the predictor roles of spiritual well-being, healthcare professionals’ support and shock anxiety in accepting ICD.

**Methods:**

This cross-sectional study was conducted on 100 patients with ICD. The data were collected by the Florida Patient Acceptance Scale, Florida Shock Anxiety Scale, Spiritual Well-Being Scale and Healthcare Professionals’ Support Questionnaire.

**Results:**

The mean (SD) scores of patient acceptance, shock anxiety, spiritual well-being and healthcare professionals’ support were 65.4 (13.56), 21.93 (8.95), 88.92 (11.78) and 76.41 (10.54), respectively. The results revealed higher acceptance among the participants with lower shock anxiety levels (*r* = −0.51, *P* < 0.001), higher mean scores of spiritual well-being (*r* = 0.33, *P* = 0.001) and higher healthcare professionals’ support (*r* = 0.40, *P* < 0.01). Additionally, the results of linear regression indicated that spiritual well-being, healthcare professionals’ support and shock anxiety predicted 36% of the patient acceptance variance (*R* = 0.61, *R*^2^ = 0.38, adj *R*^2^ = 0.36) and shock anxiety and healthcare professionals’ support were the predictors of patient acceptance.

**Conclusion:**

The study results indicated that the patients’ mean score of acceptance was relatively high. In addition, the mean scores of shock anxiety, spiritual well-being and healthcare professionals’ support were low, moderate and relatively high, respectively. Conducting healthcare professionals’ support interventions, spiritual therapy and reducing shock anxiety can help patients accept ICDs.

## Introduction

Cardiovascular diseases are the leading cause of death worldwide (1). One of the major public health concerns in the world is the increasing rate of sudden cardiac death, mostly due to life-threatening cardiac arrhythmias (2). An implantable cardioverter-defibrillator (ICD) is the first choice for patients who have survived life-threatening ventricular arrhythmias (3). ICD is used for secondary prevention among patients who have survived dangerous ventricular arrhythmias and sudden cardiac arrest. In addition, ICD is used for primary prevention in patients who already have heart diseases and are at risk of arrhythmias (2–3).

The population of patients with ICDs is increasing rapidly (4–5) because ICD placement is effective in reducing the mortality rate of sudden cardiac death and increasing the life expectancy of patients with life-threatening cardiac conditions (2). Despite the benefits of ICD, living with this device can be stressful for some recipients (6) and change their body image (7). A previous study revealed a change in these patients’ social roles and interactions (8). On the other hand, lack of sufficient information after ICD placement can lead to limited activities, feelings of concern about the future and spread of psychological problems (5), which can eventually affect patient acceptance (4). Patient acceptance means psychological adjustment with ICD and understanding its advantages and disadvantages (9). Therefore, patients with ICDs have to adjust and modify their lifestyle in order to maintain and improve their quality of life (10).

Although most patients accept ICDs, 24%–33% of them suffer from psychological distress (11) including depression and anxiety (12). An ICD shock can lead to anxiety, anger and fear (3). A previous study suggested that a significant percentage of patients might experience ICD-related anxiety or shock anxiety (6). Generally, 10%–38% of patients may experience a shock in the first year of ICD placement. Shock anxiety refers to the fear from a shock in the future and avoiding activities that may cause a shock (13). Shock anxiety is in fact one of the factors that can reduce ICD acceptance in patients (4, 14).

Another important factor in the health of patients with ICDs is their mental and emotional health. Spirituality can protect cardiac patients from emotional distress and improve their quality of life. Spiritual well-being can also protect patients with heart failure from depression (15). Spiritual well-being is so important that the World Health Organization (WHO) introduced the spiritual dimension in 1984 along with other dimensions of health such as physical, mental, and social dimensions and reported its significant impact on public health and happiness (16). Spiritual well-being has protective effects against anxiety and depression among people in a community and has positive health outcomes. The positive effects of spiritual well-being on patients’ physical and mental health have been approved, as well (17). A study suggested that patients with higher spiritual well-being had significantly lower mental distress as well as a lower prevalence of mental problems (15). Overall, spirituality can help people cope with stressful life events (16). Therefore, the role of spirituality and religion in healthcare has become increasingly important. In fact, faith plays a very important role in accepting and adapting to diseases (18).

Another factor associated with reduced ICD acceptance is the lack of awareness and knowledge of how ICD works (19). Healthcare professionals can form a support system that affects the health of patients with ICDs, because ICD placement requires the regular examination of the device usually every 3–6 months (3). Healthcare professionals play a crucial role in the process of health education and social support of patients after ICD placement. They can spend enough time to educate and support patients and help those who have experienced the anxiety of ICD shock to increase ICD acceptance (4). In this context, nurses play a key role in the process of training patients with ICDs (20). In addition to determining the physical condition, nurses should assess patients’ concerns about sexual and spiritual conditions and coping strategies (21).

A review of the literature revealed the paucity of studies evaluating the relationship between healthcare professionals’ support and shock anxiety, and acceptance among patients with ICDs (4). Additionally, only one study was found to investigate spiritual well-being amongst patients with ICDs (15). Therefore, it is worthwhile to conduct a comprehensive study using specific tools in order to measure shock anxiety, spiritual well-being, healthcare professionals’ support and ICD acceptance. The present study aims to investigate the predictor roles of spiritual well-being, healthcare professionals’ support and shock anxiety in ICD acceptance.

## Methods

### Study Design

This cross-sectional study was conducted on 100 patients with ICDs from April to August 2019.

### Setting

This study was conducted in the Heart Clinic of Kowsar Hospital and Pacemaker and ICD Clinic of Faghihi Hospital affiliated to Shiraz University of Medical Sciences, Shiraz, Iran.

### Participants

The target population included patients with ICDs registered in the heart clinic for follow-up and ICD analysis. The inclusion criteria of the study were aging 18 years old or older, speaking Persian and having had an ICD for at least 6 months. Patients with significant cognitive impairments such as dementia and pre-existing known mental illnesses and those taking psychotropic medications were excluded.

### Study Size

Based on a previous study by Morken et al. (4) reporting correlation coefficients between device acceptance and shock anxiety (*r* = −0.52), constructive support (*r* = 0.22) and non-constructive support (*r* = −0.36), considering the power of at least 80% and an error of 5%, and using the G-Power statistical software, an 81-patient sample size was estimated for the study. Finally, 100 eligible patients were enrolled.

### Data Collection Process

After the approval of the project in the Ethics Committee of Shiraz University of Medical Sciences, the necessary permissions were obtained from the hospitals’ research committees. The researcher then entered the research setting, briefed the eligible patients about the research objectives and obtained their written consent forms after they agreed to cooperate. The participants were selected via convenience sampling and were required to fill out the study questionnaires. A research assistant also accompanied the patients when they were completing the questionnaires in order to provide further explanations in case the patients had any ambiguities. If the patients did not have enough literacy, the research assistant read and explained the questionnaire items to them and completed the questionnaires according to their answers.

### Data Sources/Measurement

In this study, the data were collected using five questionnaires.

- Demographic information questionnaire: This questionnaire included age, gender, marital status, education level and occupational status. In addition, the patients were asked for information about the ICD including the duration of having an ICD, receiving shocks, total number of shocks received and type of ICD.
- Florida Patient Acceptance Scale (FPAS): This scale was first developed by Burns et al. (9) in 2005 to evaluate patients with pacemakers and ICDs. Its short form included 12 items and three subscales, namely device-related distress, positive appraisal, and return to function, each containing four items. The items were scored based on a Likert scale ranging from one (totally disagree) to five (totally agree), with higher scores representing better device acceptance (8). The total scores of acceptance and its subscales were linearly converted to a score between 0 and 100. The validity of the short form was approved by Versteeg et al. (22) and its reliability was confirmed by Cronbach’s alpha coefficient of 0.76. The short form of FPAS was used in the present study. The content and face validity of the Persian version of FPAS was approved by 10 faculty members of Shiraz University of Medical Sciences and its reliability was confirmed by Cronbach’s alpha of 0.73.
- Florida Shock Anxiety Scale (FSAS): This scale was designed by Kuhl et al. (13) for patients with ICDs in 2006 in order to assess ICD-specific anxiety and the cognitive, behavioural and emotional effects of shock. FSAS contained 10 items scored based on a 5-point Likert scale ranging from 1 (never) to 5 (always). Thus, the total score of the scale could range from 10 to 50, with higher scores indicating higher shock anxiety levels. In the original study, the validity and reliability of FSAS were confirmed and its Cronbach’s alpha coefficient was 0.91. In the present study, the content and face validity of the Persian version of the scale was confirmed by 10 faculty members of Shiraz University of Medical Sciences. In addition, Cronbach’s alpha for shock anxiety was 0.87.
- Spiritual Well-Being Scale: This scale was developed by Paloutzian and Ellison (23). It consisted of 20 items divided into two dimensions, namely religious well-being and existential well-being. The items could be scored based on a six-point Likert scale and the scores could range from 20 to 120. Accordingly, spiritual well-being was classified into three levels of low (20–40), medium (41–99) and high (100–120). The validity and reliability of the questionnaire were confirmed by Paloutzian and Ellison. Its reliability was confirmed by Cronbach’s alpha coefficient of 0.88. The validity and reliability of SWBS were approved by Soleimani et al. (24), as well. Hajian and Izadi (21) also confirmed the reliability of the scale by Cronbach’s alpha of 0.85. In the present study, the Cronbach’s alpha coefficient of the scale was computed as 0.86.
- Healthcare professionals’ support questionnaire: This questionnaire was designed by Karlsen et al. (25) in 2004 to assess healthcare professionals’ social support. Healthcare professionals include physicians, nurses and other health professionals who are involved in patients’ medical care. The initial questionnaire consisted of 11 items. The questionnaire was revised by Oftedal, Bru and Karlsen (26) by adding seven more items in 2011. In 2012, it was revised by Morken et al. (3–4) for use in studies on patients with ICDs. The final questionnaire contained 20 items including 14 items on constructive support and six on non-constructive support of healthcare professionals. These items could be scored based on a five-point Likert scale ranging from one (strongly agree) to five (strongly agree). Thus, the total score could range from 20 to 100. The reliability and validity of the questionnaire were approved by Morken et al. (3–4). Accordingly, Cronbach’s alpha coefficients were found to be 0.94 for constructive support and 0.73 for non-constructive support (3–4). In the current research, the content and face validity of the Persian version of the questionnaire was confirmed by 10 faculty members of Shiraz University of Medical Sciences. Besides, Cronbach’s alpha coefficient was calculated as 0.88, 0.88 and 0.85 for healthcare professionals’ support, constructive support and non-constructive support, respectively.

### Ethical Consideration

All study processes were conducted after gaining the approval of the Ethics Committee of Shiraz University of Medical Sciences on 28 April 2018 and based on the Declaration of Helsinki. Additionally, all participants were required to sign written consent forms. The benefits of the study were discussed in the consent form and the participants were assured about the voluntary nature of the research and their right to leave the study. Moreover, the questionnaires were coded to maintain confidentiality.

### Statistical Methods

The collected data were analysed using the SPSS for Windows (version 25). The data were reported as frequency and mean, and were analysed using Pearson’s correlation coefficient and multiple linear regression test. The significance level was set at 0.05.

## Results

### Participants

This study was conducted on 100 patients with ICDs. The participants’ mean age was 58.91 (14.09) years old (range: 20 years old–85 years old). The majority of the participants were male (71%) and married (89%). The mean duration of having an ICD was 4.83 (4.38) years and 38% of the patients had a history of receiving shocks. The majority of the patients (73%) had Medtronic ICDs. Detailed demographic and clinical variables have been presented in Table 1.

The mean score of FPAS was 65.4 (13.56), which ranged from 37 to 98. In addition, the mean scores of the device-related distress, positive appraisal, and return to function subscales were 43.61 (24.9), 87.23 (14.21) and 53.73 (17.52), respectively. The mean score of FPAS was lower in patients with shock experience (63.39 [14.17]) than in those without shock experience (66.62 [13.14]). Besides, the mean score of FPAS was lower in females (62.13 [16.01]) compared to males (66.73 [12.30]).

The mean score of FSAS was 21.93 (8.95), which ranged from 10 to 49. The mean score of anxiety disorder was higher in females (24.31 [10.48]) than in males (20.95 [8.13]). It was 22.02 (9.86) in the patients with shock experience, which was higher comparted to the patients without shock experience (21.87 [8.43]).

The mean score of spiritual well-being was 88.92 (11.78), which ranged from 45 to 119. The mean score of religious well-being (46.88 [6.10]) was higher than that of existential well-being (42.04 [7.08]). Furthermore, most of the participants (86%) enjoyed moderate levels of spiritual well-being and none was in the low range category.

The mean score of healthcare professionals’ support was 76.41 (10.54), which ranged from 45 to 98. The mean scores of constructive and non-constructive support were 53.27 (8.45) and 12.86 (4.26), respectively.

As shown in Table 2, a significant correlation was found between patient acceptance and shock anxiety (*r* = −0.51, *P* < 0.001), spiritual well-being (*r* = 0.33, *P* = 0.001) and healthcare professionals’ support (*r* = 0.40, *P* < 0.001). Accordingly, higher acceptance was accompanied by higher scores of spiritual well-being and healthcare professionals’ support and lower scores of shock anxiety. The results also showed a significant positive correlation between patient acceptance and constructive healthcare professionals’ support (*r* = 0.26, *P* = 0.009) and a significant negative relationship between patient acceptance and non-constructive healthcare professionals’ support (*r* = −0.49, *P* < 0.001).

According to the results of linear regression, the three subscales of spiritual well-being, healthcare professionals’ support and shock anxiety predicted 36% of patient acceptance variance (*R* = 0.61, *R^2^* = 0.38, adj *R^2^* = 0.36). The results of linear regression presented in Table 3 also showed that patient acceptance was correlated to shock anxiety and healthcare professionals’ support. In other words, shock anxiety followed by healthcare professionals’ support predicted patient acceptance. Accordingly, lower shock anxiety and higher healthcare professionals’ support for patients with ICDs increased their acceptance. However, spiritual well-being did not predict patient acceptance in this study. Based on the regression coefficient column, the regression can be presented as follows:


$$\begin{array}{l} \text{Patient acceptance}=39.40+0.32\times \\ \text{healthcare }\text{professionals}’\;\text{support}+0.17\times \\ \text{spiritual well-being}-0.65\times \text{shock anxiety} \end{array}$$

## Discussion

The study results indicated that shock anxiety and healthcare professionals’ support were the predictors of patient acceptance. Patient acceptance is important, because higher device acceptance improves their quality of life and reduces their psychological distress (22). In the present study, the mean score of device acceptance (FPAS) was 65, which was similar to the results of the research by Chair et al. (27) but lower than the acceptance rate in some other studies (4, 19, 22). Moreover, the female participants’ mean score of acceptance was lower compared to the male participants. The individuals with a history of shock also had a lower acceptance rate. In several studies, female sex (22, 27–28) and a history of having shocks (4) were associated with lower ICD acceptance.

In the current research, the mean score of shock anxiety was 22, which was similar to the results of the research carried out by Richards, Kramer and Sears (14) but higher than the mean score of shock anxiety in some studies (6, 13, 27, 29). It is worth mentioning that there was no shock anxiety classification in the study by Richards, Kramer and Sears (14) but Tripp et al. (29) updated and classified individuals’ anxiety based on the shock anxiety scores. In this way, target groups can be identified for therapeutic-psychological measures and programmes. For instance, people with the shock anxiety scores of 21–30 have mild anxiety symptoms and, consequently, should be provided with the necessary trainings on the purpose of ICD placement and the likelihood of shock (29). The shock anxiety level in patients can be influenced by a variety of factors including race and gender. Females, for example, usually have higher shock anxiety levels (27, 29). Such factors as receiving shocks (6, 14), increased number of shocks, and recent shocks also increase shock anxiety amongst patients (30). In the current research, the shock anxiety level was higher in females as well as in the participants with a history of having shocks.

The results of the present study demonstrated that most patients had moderate levels of spiritual well-being. They also had higher levels of religious well-being in comparison to existential well-being. Although many studies have assessed spiritual well-being (15, 17, 24, 31), no studies were found to evaluate spiritual well-being using the SWBS in patients with ICDs. Musavi et al. (31) reported a moderate level of well-being in the majority of Iranian haemodialysis patients. The results of another study revealed a higher-than-average level of spiritual well-being among cardiac patients (32). Yet, the mean score of spiritual well-being was higher in the present study compared to the other studies conducted on the issue (17, 31–32).

The healthcare professionals’ support score in the present study was 76, which represented the relatively high support of healthcare professionals. Although Morken et al. (3, 4) used healthcare professionals’ support scale in two studies, they did not mention the mean scores of the numerical scales.

In the current study, shock anxiety and healthcare professionals’ support were the predictors of patient acceptance. Accordingly, reduction in shock anxiety and increase in healthcare professionals’ support improved patient acceptance. Although spiritual well-being has been found to be effective in reducing psychological distress (15) and increasing the ability to cope with stressful live events (16), it was not a predictor of patient acceptance in this study. Wilson et al. (19) also reported that shock anxiety, depression and knowledge about the device were the most important predictors of device acceptance. Similarly, Richards, Kramer and Sears (14) investigated the predictors of shock anxiety in patients with ICDs and reported that patient acceptance, social support, and receiving shocks were the most important factors influencing shock anxiety. Moreover, the results of some studies suggested that cognitive and behavioural interventions in patients could reduce shock anxiety (29). In the present study, a significant correlation was observed between healthcare professionals’ support and shock anxiety reduction. Thus, medical staff might be able to help reduce anxiety by educating and supporting patients.

The present study findings indicated that the patients perceived a relatively high support from healthcare professionals. Providing constructive support such as spending time to explain about heart diseases and ICD and listening to patients’ concerns on the part of healthcare professionals had a considerable effect on patient acceptance. However, providing non-constructive support by healthcare professionals including the provision of information that could lead to the feelings of fear and skepticism in patients was associated with lower acceptance. In the same line, Morken et al. (4) stated that constructive support and patient education could enhance their positive attitudes towards ICD, thereby improving acceptance. Sensitivity in communication with patients, spending enough time for them, and not providing them with information that could lead to their misinterpretation could be effective in reducing non-constructive support. Pedersen et al. (33) maintained that face-to-face discussions with patients prior to ICD placement would allow healthcare staff to identify patients’ distress and to respond to their concerns. The results of a study performed on Singaporean patients with ICDs also showed that healthcare professionals’ support could lead to higher patient acceptance (11). However, both healthcare professionals and patients believed that more time should be devoted to educating patients during admission (34).

Since increased shock anxiety and reduced healthcare professionals’ support were associated with lower patient acceptance, healthcare professionals are recommended to increase their support for patients in an informative, emotional, and instrumental manner to increase their acceptance.

### Limitation

The cross-sectional design of this study was one of its limitations. Thus, future prospective and long-term studies are warranted. In addition, further studies are recommended to investigate the effect of supportive interventions by healthcare professionals on shock anxiety and acceptance of patients with ICDs.

### Generalisability

In order to increase the generalisability of the findings, further studies with larger sample sizes are recommended.

## Conclusion

The results of the present study showed the patients’ relatively high mean score of acceptance. Meanwhile, the mean scores of shock anxiety, spiritual well-being and healthcare professionals’ support were low, moderate and relatively high, respectively. Moreover, the results revealed higher acceptance among the individuals who had lower shock anxiety levels as well as those with higher mean scores of spiritual well-being and healthcare professionals’ support. The results of linear regression also indicated that shock anxiety and healthcare professionals’ support were the predictors of patient acceptance.

## Figures and Tables

**Table 1:** Demographic and clinical characteristics of the patients with ICDs (*n* = 100)

Variables	*n* (%)^a^,^b^
Sex	
Male	71
Female	29
Having ICD^c^ shock experience	
Yes	38
No	62
Marital status	
Married	89
Single	6
Divorced	1
Widowed	4
Education level	
Illiterate	34
Secondary school	36
High school diploma	23
Bachelor’s degree	7
Occupation	
Employed	17
Unemployed	14
Retired	31
Disabled	14
Homemaker	24

Notes:

a Number (*n*) is the same as percentage (%);

b Frequency and %;

c ICD = implantable cardioverter-defibrillator

**Table 2:** Pearson’s correlation coefficients among FPAS, FSAS, SWB, healthcare professionals’ support and patient acceptance subscales

	FPAS^a^/device acceptance *r (P-*value)^a^	Spiritual well-being *r* (*P-*value)	Healthcare professionals’ support *r* (*P-*value)
FSAS^b^	−0.51 (< 0.001)**	−0.17 (0.001)	−0.21 (< 0.001)*
FPAS^c^ subscales			
Device-related distress	−0.82 (< 0.001)**	−0.34 (< 0.001)**	−0.30 (0.002)**
Return to function	0.78 (< 0.001)**	0.13 (0.19)	0.28 (0.004)**
Positive appraisal	0.45 (< 0.001)**	0.21 (0.03)*	0.29 (0.004)**
Spiritual well-being	0.33 (0.001)**	1	0.43 (< 0.001)**
Healthcare professionals’ support	0.40 (< 0.001)**	0.43 (< 0.001)**	1

Notes:

a Correlation coefficient (*r*);

b Florida Shock Anxiety Scale (FSAS);

c Florida Patient Acceptance Survey (FPAS);

** Correlation is significant at the 0.01 level;

* Correlation is significant at the 0.05 level

**Table 3:** Multiple linear regression analysis predicting patient acceptance (*n* = 100)

Factors^a^	Adjusted (95% CI)^b^	*P*-value^c^
1 (constant)	39.49 (18.04, 60.94)	< 0.001
Healthcare professionals’ support	0.32 (0.09, 0.55)	0.007
Spiritual well-being	0.17 (−0.02, 0.38)	0.088
Shock anxiety	−0.65 (−0.90, −0.41)	< 0.001

Notes:

a Dependent variable: device acceptance;

b Adjusted regression coefficient;

c Multiple linear regression (*R*^2^ = 0.36)
